# Supplementary material for: Risk factors for mechanical ventilation and ECMO in COVID-19 patients admitted to the ICU: A multicenter retrospective observational study
Source: PLoS One. 2022 Nov 14;17(11):e0277641. doi: 10.1371/journal.pone.0277641 (PMC9662741; doi:10.1371/journal.pone.0277641)
Supplement: S1 Table — Data are presented as the median (interquartile range) or number (%). Other medications included hydroxychloroquine, favipiravir and lopinavir-ritonavir. The total number of patients receiving renal replacement therapy indicates the number of patients who underwent hemodialysis and/or continuous hemodiafiltration. † Mechanical ventilation-only vs. ECMO group, P < 0.01, Mann-Whitney U test. Patients who died were excluded from the analysis. ‡ Two patients in the ECMO group who were intubated before ICU admission were excluded from the analysis. HD, hemodialysis; CHDF, continuous hemodiafiltration; NA, not applicable. (DOCX) [file pone.0277641.s001.docx]

|  | All (n=66) | No mechanical ventilation (n=24) | Mechanical ventilation-only (n=22) | ECMO (n=20) |
| --- | --- | --- | --- | --- |
| **Medications for COVID-19** |  |  |  |  |
| Remdesivir | 26 (39.4%) | 3 (12.5%) | 10 (45.5%) | 13 (65.0%) |
| Dexamethasone | 51 (77.3%) | 16 (66.7%) | 19 (86.4%) | 16 (80.0%) |
| Others * | 27 (40.9%) | 12 (50.0%) | 9 (40.9%) | 6 (30.0%) |
| **Renal replacement therapy** |  |  |  |  |
| Hemodialysis | 13 (19.7%) | 3 (12.5%) | 3 (13.6%) | 7 (35.0%) |
| Continuous hemodiafiltration | 15 (22.7%) | 0 (0%) | 5 (22.7%) | 10 (50.0%) |
| Total | 19 (28.8%) | 3 (12.5%) | 6 (27.3%) | 10 (50.0%) |
| **Airway management** |  |  |  |  |
| Duration of mechanical ventilation | 18.0 (11.5-32.0) | NA | 11.5 (8.0-21.3) | 29.0 (18.0-36.0) † |
| Time from symptom appearance to the introduction of mechanical ventilation | 7.5 (5.0-9.8) | NA | 8.0 (5.3-11.0) | 6.5 (4.8-8.3) |
| Time from ICU admission to the introduction of mechanical ventilation | 0 (0-1.0) | NA | 0 (0-0.8) | 0 (0-1.0) ‡ |
| Tracheotomy | 14 (21.2%) | NA | 7 (31.8%) | 7 (35.0%) |
| Time from the introduction of mechanical ventilation to tracheotomy | 25.0 (19.3-32.5) | NA | 24.0 (16.5-26.5) | 27.0 (22.0-38.0) |

**S1 Table**
