# Supplementary material for: Risk factors for mechanical ventilation and ECMO in COVID-19 patients admitted to the ICU: A multicenter retrospective observational study
Source: PLoS One. 2022 Nov 14;17(11):e0277641. doi: 10.1371/journal.pone.0277641 (PMC9662741; doi:10.1371/journal.pone.0277641)
Supplement: Attachment — Submitted filename: Response to Reviewers 101922ver2.docx [file pone.0277641.s003.docx]

Comments of Reviewer 1
Manuscript is a clear, concise, and well-written with relevant introduction.

Conclusion in abstract should be written more clearly and precisely.

Reply: We have modified the conclusion in the abstract according to your suggestion.

In Methodology explain terms APACHE II score, SOFA score, and how did you calculate them.

Reply: We have added a description of APACHE II and SOFA scores in the Methods section Further, we have cited references for where more information on these scores can be obtained.

Some values in Table 1 and others are express as numbers and percentages, please write n(%) for that variables.

Reply: We have changed the display of the variables in Table 1, as recommended.

If it is possible, please divide Table 2 and Table 3 into two or more tables.

Reply: Tables 2 and 3 are certainly large, but we would like to present the findings at a glance. Hence, we would prefer to retain the table in its current form, if there are no space constraints according to the journal requirements.

Comments of Reviewer 2
The manuscript PONE-D-22-26070 is a well prepared original article about risk factors of intensive care either with ECMO or mechanical ventilation in Covid-19 patients. I congratulate authors for such a good work. My comments about the sections of the manuscript are as following:
Title is relevant as keywords after abstract which is an adequate summary of the study.
Rationale and aims are clearly mentioned in introduction
Methods and the study design expressed perfectly. Statistical analyses are correct and adequate for the study design.
Presentation of the results is clear. Tables improved the readiness. However, unit of all variables must be stated in the tables. I advise n,% for categorical variables.

Reply: We have mentioned the units of the variables in Table 1, as recommended.

Discussion is fair enough. Yet, I suggest discussing the role of inflammatory burden on necessity of ECMO or mechanical ventilation. Studies in literature reported higher inflammatory markers in serious Covid-19 cases compared to the patients with mild or moderate disease (Hematology 2021;26(1):529-542. DOI: 10.1080/16078454.2021.1950898).

Reply: Thank you for presenting this important article. The article emphasizes a decrease in lymphocytes as an indicator of COVID-19 severity, especially when compared to neutrophils and platelets. We have cited this article as Reference 9.

Conclusions must be clarified. The statement 'This study revealed several risk factors on ICU admission that are related to the 261 need for subsequent MV or ECMO in COVID-19 patients admitted to the ICU.' is not the best conclusion that could be drawn from the study.

Reply: We have modified the conclusion according to your suggestion.

Journal requirements:

When submitting your revision, we need you to address these additional requirements.

1. Please ensure that your manuscript meets PLOS ONE's style requirements, including those for file naming. The PLOS ONE style templates can be found at

https://journals.plos.org/plosone/s/file?id=wjVg/PLOSOne_formatting_sample_main_body.pdf and https://journals.plos.org/plosone/s/file?id=ba62/PLOSOne_formatting_sample_title_authors_affiliations.pdf

Reply: We have made revisions to ensure that the manuscript matches the style of PLOS ONE.

2. Please provide additional details regarding participant consent. In the ethics statement in the Methods and online submission information, please ensure that you have specified what type you obtained (for instance, written or verbal, and if verbal, how it was documented and witnessed). If your study included minors, state whether you obtained consent from parents or guardians. If the need for consent was waived by the ethics committee, please include this information.

Reply: We have added the following sentence as additional details regarding participant consent: Since the ethics committees of the participating institutions waived the need for obtaining verbal or written consent from the patients participating in this study, patient informed consent was obtained in the form of the option to opt-out on the institutions’ websites.

The current study did not include minors.

3. In your Data Availability statement, you have not specified where the minimal data set underlying the results described in your manuscript can be found. PLOS defines a study's minimal data set as the underlying data used to reach the conclusions drawn in the manuscript and any additional data required to replicate the reported study findings in their entirety. All PLOS journals require that the minimal data set be made fully available. For more information about our data policy, please see http://journals.plos.org/plosone/s/data-availability.

Upon re-submitting your revised manuscript, please upload your study’s minimal underlying data set as either Supporting Information files or to a stable, public repository and include the relevant URLs, DOIs, or accession numbers within your revised cover letter. For a list of acceptable repositories, please see http://journals.plos.org/plosone/s/data-availability#loc-recommended-repositories. Any potentially identifying patient information must be fully anonymized.

Important: If there are ethical or legal restrictions to sharing your data publicly, please explain these restrictions in detail. Please see our guidelines for more information on what we consider unacceptable restrictions to publicly sharing data: http://journals.plos.org/plosone/s/data-availability#loc-unacceptable-data-access-restrictions. Note that it is not acceptable for the authors to be the sole named individuals responsible for ensuring data access.

We will update your Data Availability statement to reflect the information you provide in your cover letter.

Reply: In this revision, we provide the minimal underlying dataset of the study as Supplementary Table S2. The table contains anonymized patient data.

4. Please amend either the title on the online submission form (via Edit Submission) or the title in the manuscript so that they are identical.

Reply: We confirmed that the title on the online submission form (via Edit Submission) and the title in the manuscript are identical.

5. Your ethics statement should only appear in the Methods section of your manuscript. If your ethics statement is written in any section besides the Methods, please delete it from any other section.

Reply: We have removed the ethics statement apart from that in the methods section.

6. Please review your reference list to ensure that it is complete and correct. If you have cited papers that have been retracted, please include the rationale for doing so in the manuscript text, or remove these references and replace them with relevant current references. Any changes to the reference list should be mentioned in the rebuttal letter that accompanies your revised manuscript. If you need to cite a retracted article, indicate the article’s retracted status in the References list and also include a citation and full reference for the retraction notice.

Reply: We have confirmed that the reference list is complete and correct. The references we have cited do not include retracted articles.
